# Clinical validation of kinematic assessments of post-stroke upper limb movements with a multi-joint arm exoskeleton

**DOI:** 10.1186/s12984-021-00875-7

**Published:** 2021-06-02

**Authors:** Florian Grimm, Jelena Kraugmann, Georgios Naros, Alireza Gharabaghi

**Affiliations:** Department of Neurosurgery and Neurotechnology, Institute for Neuromodulation and Neurotechnology, University Hospital and University of Tübingen, Otfried-Mueller-Str. 45, 72076 Tübingen, Germany

**Keywords:** Human–machine interface, Exoskeleton, Sensorimotor interaction, Virtual reality, Hand-arm model, Movement analysis, Rehabilitation robotics, Neurorehabilitation, Stroke

## Abstract

**Background:**

The clinical evaluation of the upper limb of severely impaired stroke patient is challenging. Sensor-based assessments may allow for an objective evaluation of this patient population. This study investigated the validity of a device-assisted approach in comparison to the clinical outcome that it is supposed to reflect.

**Methods:**

In nineteen severely impaired chronic stroke patients, we applied a gravity-compensating, multi-joint arm exoskeleton (Armeo Spring) and compared this sensor-based assessment with the clinical outcome measure Upper Extremity Fugl-Meyer Assessment (UE-FMA) scale. Specifically, we assessed separately and subsequently the range of motion in joint space for four single joints (i.e., wrist, elbow and shoulder flexion/extension (FE), and shoulder internal/external rotation (IER)), and the closing and opening of the hand with a pressure sensor placed in the handle.

**Results:**

Within the kinematic parameters, a strong correlation was observed between wrist and elbow FE (r > 0.7, p < 0.003; Bonferroni corrected). The UE-FMA was significantly predicted by a multiple regression model (F (5, 13) = 12.22, p < 0.0005, adj. *R*^*2*^ = 0.83). Both shoulder IER and grip pressure added significantly (p < 0.05) to the prediction with the standardized coefficients β of 0.55 and 0.38, respectively.

**Conclusions:**

By applying an exoskeleton-based self-contained evaluation of single-joint movements, a clinically valid assessment of the upper limb range of motion in severely impaired stroke patients is feasible. Shoulder IER contributed most relevantly to the prediction of the clinical status. These findings need to be confirmed in a large, independent patient cohort.

## Background

Currently, the majority of stroke patients will not regain full function of the affected upper limb [1, 2]. This impairment is a decisive factor for their diminished quality of life [3]. Early and high-dose movement therapies are relevant for clinically meaningful improvements [4]. Furthermore, the assessment of upper limb movements is crucial in monitoring and understanding sensorimotor recovery [5]. An increase in the assessment frequency by means of kinematic parameters could, therefore, optimize clinical assessment procedures and enhance the effectiveness of rehabilitation treatments [6]. Particularly in severely impaired stroke patients, objective assessments are necessary to identify even small improvements in the course of a therapeutic intervention.

Such movement data may be acquired by various mechanical or optical systems, e.g., CyberGlove [7], orthotic exoskeletons [8–14], gaming systems [15–17], or in combination with robotic systems for haptic feedback such as Rutgers Master II-ND haptic glove, MIT-Manus [18] or ARMIN [19]. Devices such as the Armeo Spring [8–14], Armeo Power [20], ARMIN [19], Pneu-Wrex [21], ULEXO7 [22], ANYexo [23] and Harmony [24] have the advantage of providing at least partial kinematic registration of the upper limb movement for different joints. By contrast, systems such as the MIT-Manus [18], ReaPLAN [25, 26], ReoGo [27], Planar robot [27], and PUPArm [28] allow for endpoint-based alignment. With these latter devices, the movement of the shoulder and upper arm is estimated as a surrogate parameter, and not directly via sensors. However, such indirect measurements may miss small improvements in severely impaired patients.

There is a large variety of kinematic parameters that have been applied for upper limb evaluations such as movement accuracy, efficacy, planning, precision, smoothness, speed, spatial and temporal posture; some of them have also been correlated to clinical outcome measures following stroke [6, 29, 30], while the Upper-Extremity Fugl–Meyer Assessment (UE-FMA) scale [31] was most frequently being applied for the estimation of the clinical impairment level [32]. A number of kinematic parameters showed a significant association with the clinical evaluation (correlation coefficient of more than 0.7); however, the majority of the kinematic parameters showed either weak (less than 0.3) or moderate (0.3–0.7) associations [6]. This limited association may be related to the fact that kinematic measurements of the proximal component of the upper limb are often missing [3].

Most of the applied kinematic parameters resulted from rather complex training exercises and were not acquired for each upper limb segment separately. Specifically, either 2D pointing, 2D shape drawing, 3D pointing or 3D reach-to-grasp tasks were performed first, and then a posthoc segmental evaluation was conducted, e.g., of angle data for shoulder movement [33], range of arm elevation [15], elbow flexion/extension (FE) [33], and wrist FE [15–17]. These previous approaches are, however, at odds with the most recent systematic review on kinematic assessments of upper limb movements after stroke [29]. It suggested that the measures should be acquired with the help of a self-contained task and not during the exercises that the patient is doing for rehabilitation training, since the latter would confound the results for upper limb evaluation by including exercise-specific learning effects [34].

In this context, the first study that quantified the active range of motion for each segment separately found overall promising correlations to motor function [17], but did not use the clinical gold standard measure UE-FMA scale for this purpose. For the evaluation of severely impaired stroke patients, who are often not able to move the upper limb against gravity, the assessment device would, furthermore, need to balance gravity and capture even small movements of single joints. Therefore, multi-joint exoskeletons such as the passive Armeo Spring [8, 9, 11, 12] or the active (i.e., robotic) Armeo Power [20] are suitable for this purpose. These exoskeletons show high interaction forces between the measurement system and patient due to friction, inertia and arm weight support. More recent devices reduce friction by actuator choice, and lower inertia by lightweight design and the use of interaction force sensors [23, 24, 35, 36]. However, exoskeleton-based assessment tools necessitate a systematic evaluation to estimate their clinical validity. Specifically, there is currently no study that assessed the upper limb of severely impaired stroke patients with a multi-joint exoskeleton in comparison to the UE-FMA scale. The present study intended to close this gap.

## Methods

We recruited 19 stroke patients (8 females, mean age: 56 ± 11 [from 34 to 71] years) in the chronic phase after stroke (78 ± 55 [from 8 to 244] months) who presented with severe and persistent hemiparesis (13 right-sided, 6 left-sided; 11 ischemic, 8 hemorrhagic) and who provided written informed consent (for demographic information see Table 1). Patient inclusion criteria were: age ≥ 18 years, time since stroke: ≥6 months, UE-FMA: ≤30 out of 66 points. Participants were excluded from the study if they had uncontrolled epilepsy, drug abuse, psychiatric diseases, a bilateral motor deficit, a severe and uncontrolled clinical disease, cognitive impairment, pregnancy, metal implants or a cardiac pacemaker.

The UE-FMA captures the motor function and contains the subscores A (upper extremity), B (wrist), C (hand) and D (coordination/speed), resulting in a total of max. 66 points. This clinical evaluation was performed by two examiners at the same time to minimize assessment variability. Clinical and kinematic assessments were done subsequently. The average UE-FMA score of the whole patient group was 16.1 ± 5.2 points; the individual patient scores had a range from 7 to 29 points; thus, the study included only severely impaired patients. This study was approved by the ethical review committee of the local medical faculty.

**Table 1:** Demographic information for all participants

Patient no.	Age	Type of stroke	Gender	Side of stroke	Month post-stroke	UE-FMA
1	56	Hemorrhagic	Male	Right	56	29
2	52	Ischemic	Male	Right	156	22
3	68	Hemorrhagic	Male	Right	34	16
4	55	Hemorrhagic	Male	Right	88	10
5	67	Hemorrhagic	Male	Right	75	7
6	69	Ischemic	Female	Right	130	16
7	69	Ischemic	Male	Right	81	14
8	34	Hemorrhagic	Male	Right	45	13
9	63	Ischemic	Female	Right	58	16
10	59	Ischemic	Female	Left	20	19
11	63	Ischemic	Female	Left	133	13
12	51	Ischemic	Female	Right	21	22
13	56	Ischemic	Female	Right	87	22
14	49	Hemorrhagic	Male	Left	69	21
15	71	Hemorrhagic	Male	Right	244	14
16	41	Hemorrhagic	Male	Right	62	9
17	48	Ischemic	Male	Left	8	13
18	36	Ischemic	Female	Left	32	16
19	49	Ischemic	Female	Left	81	14

### Exoskeleton and visualization

The basic methodology of our exoskeleton-based training and assessment setup has already been described in detail in previous studies and is cited here accordingly [8, 9, 37]. We used a commercially available (Armeo Spring, Hocoma, Volketswil, Switzerland) rehabilitation exoskeleton with separate sensors for shoulder (arm rotation, arm elevation), elbow (FE) and wrist joints (FE, pronation/supination) to provide gravity-balancing support for the paretic arm and simultaneous registration of movement kinematics and grip force.

This device enabled us to make individual adjustments of gravity compensation, thereby supporting patients with severe impairment in performing task-oriented practice within a motivating virtual environment. To align posture and to minimize the exoskeleton-patient interaction, the same position (neutral zero) with a distance of 90 degrees between forearm and upper arm, with the shoulder being adducted to the trunk and with the thumb pointing upwards, was applied as the starting position for all assessments. In accordance with the manufacturer’s instructions, the length of the different components of the exoskeleton with regard to the wrist, forearm and upper arm was adjusted to suit the individual anatomical proportions of each patient. Gravity compensation was set according to the manufacturer’s instruction, thereby, allowing for a complete gravity compensation of the upper limb in the neutral zero position. In this context, a better understanding of the weight compensation provided by this device may help to fully utilize it in clinical and research settings [10]. A file mapping communication protocol was used to read the real-time movement data, as originally represented in the angles of all arm joints, and the grip force measured by the device from a shared memory block.

Using the real-time sensor data of the exoskeleton to display a three-dimensional multi-joint visualization of the user’s arm in virtual reality (VR), we extended these features in-house to provide both visual and auditory instructions and feedback for the patient. Since our exoskeleton-based rehabilitation interventions were already using this VR set-up, we applied the same technology also for the assessment protocol to avoid a methodological disruption of the integrated training and assessment sessions. The aim of this VR approach was, furthermore, to standardize the evaluation independent of the interaction of an examiner to reduce assessment variability. The system’s features allow for further optimization (e.g., multimodal feedback, personalized content, gamification) in the future. The real-time sensor data enabled us to display a natural virtual representation of the patient’s arm on a computer screen. This provides the patient with additional visual feedback on how the movement was performed. The virtual arm engine was programmed in a Microsoft XNA framework. The arm model utilized by the engine was constructed as a meshed bone-skin combination with 56 bones which were modelled as interconnected bodies in the simulation (3Ds Max 2010TM, Autodesk). This model included 14 finger bones, 11 hand route bones and one bone for each shoulder, forearm and upper arm for each side of the body [9]. The real-time sensor data modulated the 3D model displayed on a 2D screen. Specifically, the joint angles and grip forces of the device measured with the exoskeleton were used to modify the pose of the bones (i.e., position of the bone objects in CAD space) of the meshed model in accordance with the movements of the user, thereby providing online closed-loop feedback. The joint angles of the exoskeleton were directly represented in virtual reality, while the grip forces were amplified to feedback natural hand function.

### Movement assessment design

The positioning of the patient in the exoskeleton (~ 5 min), including the complete movement assessment (~ 6 min) and the clinical evaluation with the UE-FMA score (~ 30 min) were performed on the same day. Prior to the examination, the patients were instructed by the examiner on how to perform the movements for the assessment. To facilitate an efficient evaluation of the motor abilities of severely affected stroke patients, the kinematic registration of the active range of motion of the impaired arm was conducted in one self-contained session, i.e., the task was different and separated from the tasks in the training sessions.

**Fig. 1:**
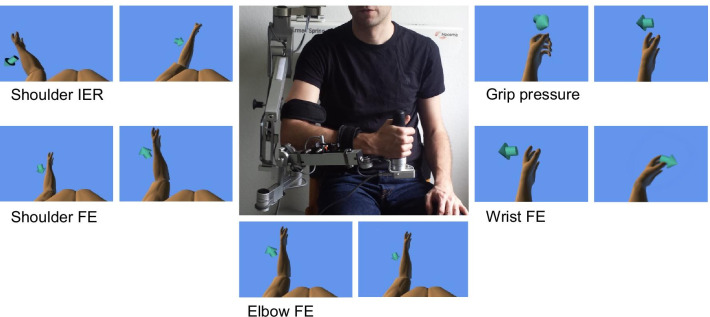
Subject with exoskeleton (in the center); visualization of the instructed movements of the five assessment tasks. The required direction of movement is indicated by a text (e.g., flex/exted the wrist) and by arrows. The actual movement of the patient is displayed as real-time feedback with a 2D projection of a 3D avatar

A software design instructed the patients by arrows, text that indicated the respective instructions (e.g., flex/extend the wrist) and tone messages to repeat single-joint movements while providing feedback related to the performed movements and the range of motion (Fig. 1). The simple instructions and single-joint movements ensured that the self-contained movements could be performed by patients of all cognitive levels. Since these tasks were designed to measure the maximum range of motion of single-joints in joint space, reference points did not require tracking and so no overshoots occurred, which otherwise may be observed during 3D motion tracking when 3D rendering is displayed on a 2D screen [38]. In this study, we designed simple, self-contained tasks that minimize patient-exoskeleton interactions and do not rely on learning [29], thus preventing potential confounds that are related to human-device interactions but not to motor recovery [34].

In order to develop a fast and practical assessment for severely impaired stroke patients that could be applied in the context of daily rehabilitation sessions, the overall number of evaluated parameters was restricted. The single-joint movements of this task were, moreover, carefully selected to be independent of the exoskeleton environment, i.e., they could also be translated to the environment outside the exoskeleton if the patients’ training progress eventually enabled them to perform them without gravity-compensation. The following joints were measured subsequently and selectively: grip pressure (difference between closing and opening the hand), wrist FE, elbow FE, shoulder FE and shoulder internal/external rotation (IER); shoulder ab- and adduction was limited due to the physical constraints of the exoskeleton and was not evaluated. Also, pronation/supination was not assessed in this study. During each joint movement, the other joints were blocked to measure improvements without compensatory movements. Each task was performed 5 times, allowing the movement to be performed for 5 s in each direction followed by a 5 s rest period.

All joint movement data for the wrist, elbow, upper arm, and shoulder were recorded during the exercises in °. The grip pressure was estimated in kilopascal (kPa).

### Statistics and data evaluation

Statistical analysis was performed on a Matlab 2010b Engine and SPSS (IBM SPSS Statistics for Windows, Version 22.0. Armonk, NY: IBM Corp.). The extent of the kinematic parameters was calculated as a mean over the trials.

A multiple regression was performed to predict the UE-FMA score from grip pressure, wrist FE, elbow FE, shoulder FE, and shoulder IER. The linearity was assessed by partial regression plots and a plot of studentized residuals against the predicted values. The independence of residuals was assessed by a Durbin–Watson statistic. The assumption of normality was assessed by a Q–Q Plot. The significance level was set at p = 0.05 for all tests.

A Pearson’s product–moment correlation was estimated to assess the relationship within and between the subscores A–D of the UE-FMA and the kinematic parameters. The analyses showed a linear relationship with the variables being normally distributed as assessed by the Shapiro–Wilk test (*p* > 0.05); there were no outliers.

## Results

Patients became easily accustomed to the assessment setup without adverse effects. The system and the training-software ran smoothly throughout the evaluation. Due to the gravity compensation of the exoskeleton, all severely impaired patients were able to perform the movement assessment task.

### Kinematic parameters

**Fig. 2:**
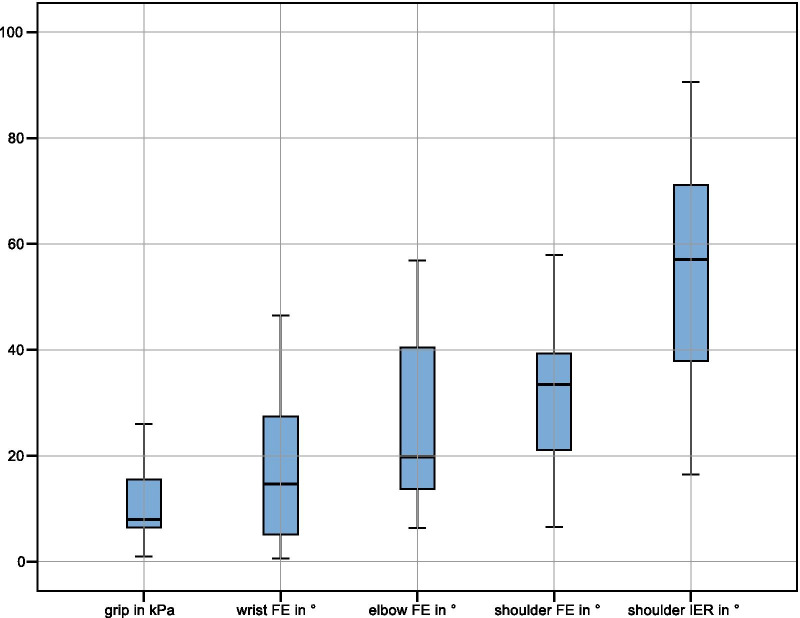
Boxplots of kinematic assessment results of all patients (FE: flexion/extension, IER: internal/external rotation). Displayed is the mean relative range of motion in joint space for wrist FE, elbow FE, shoulder FE and shoulder IER, and grip pressure, for all patients. According to literature, the physiological grip strength [39] and active range of motion [40] of healthy subjects are as follows: Medial grip strength: 53 kPa, wrist FE: 144°, elbow FE: 146°, shoulder FE: 158°, shoulder IRE: 160°. The device maxima are: grip: 93 kPa, wrist FE: 180°, elbow FE: 90°, shoulder FE: 90°, shoulder IER: 180°+

**Fig. 3:**
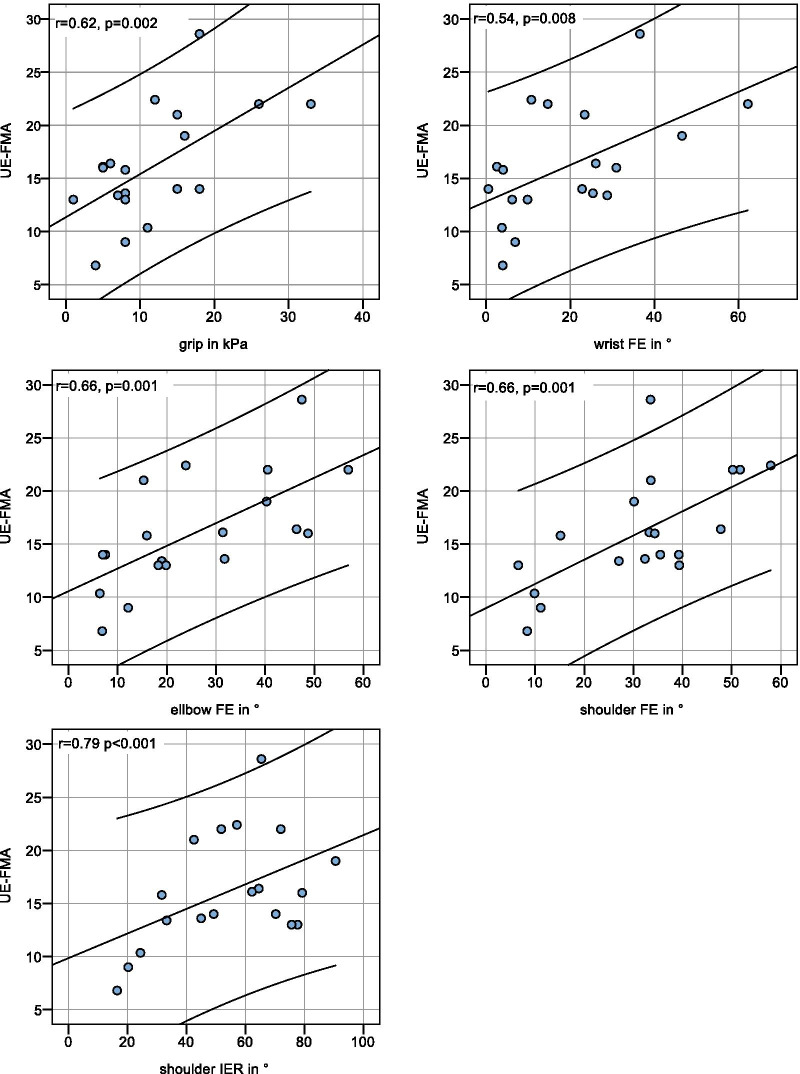
Scatter plots with Pearson correlation between clinical (UE-FMA total score) and kinematic parameters (grip force, wrist FE, elbow FE, shoulder FE and IER). The regression line with r and p-value is displayed with the 95 % confidence intervals

**Fig. 4:**
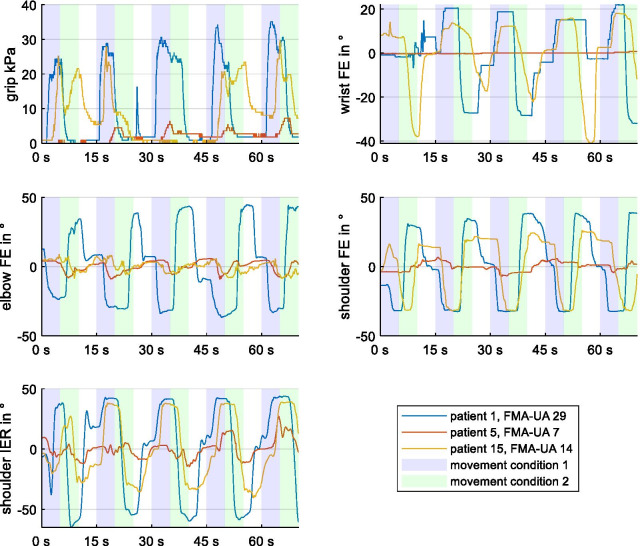
Kinematic data. Real-time data of individual movements for the angle data and pressure values are displayed for three exemplary patients (1, 5 and 15) for the five tasks. Marked are the movement conditions 1 (blue) and 2 (green) and the resting condition (white). The plots indicate amplitude differences between the different patients for the movement conditions (1/2): grip/release, wrist FE, elbow FE, shoulder FE, shoulder outside/inside rotation

On average, 6 min and 15 s were required to register the patients’ movement abilities. One operator (who positioned the patients in the exoskeleton) was present during the assessment but did not need to intervene in the evaluation procedure since the instructions, feedback and exercises ran smoothly. The ensuing kinematic performance parameters are presented in Fig. 2, and their correlations with the UE-FMA score in Fig. 3. Exemplary real-time kinematic data of three patients is illustrated in Fig. 4.

Within the kinematic parameters, there was a strong correlation between wrist and elbow FEs (r > 0.7, p < 0.003; Bonferroni corrected, Table 2). Between the kinematic and clinical parameters, there was a moderate to high correlation between all instrumental measures and the UE-FMA subscores A (upper extremity) and D (coordination/speed)

**Table 2:** Cross-correlation of kinematic parameters and correlations with the UE-FMA sub scores A-D (r, upper section) and corresponding p-values (p, lower section)

		Grip	Wrist FE	Elbow FE	Shoulder FE	Shoulder IER
r	Grip in kPa	1.000	0.394	0.348	0.463	0.254
	Wrist FE in °	0.394	1.000	0.735*	0.422	0.475
	Elbow FE in °	0.348	0.735*	1.000	0.547*	0.614*
	Shoulder FE in °	0.463	0.422	0.547*	1.000	0.543
	Shoulder IER in °	0.267	0.475	0.614	0.543	1.000
	UE-FMA total	0.621*	0.543*	0.658*	0.662*	0.788*
	UE-FMA subscore A	0.624*	0.618*	0.790*	0.725*	0.794*
	UE-FMA subscore B	0.434	0.187	0.17	0.334	0.427
	UE-FMA subscore C	0.120	0.043	0.120	0.119	0.415
	UE-FMA subscore D	0.593*	0.605*	0.718*	0.585*	0.684*
p	Grip in kPa	0.0	0.048	0.072	0.023	0.295
	Wrist FE in °	0.048	0.0	**0.000**	0.036	0.040
	Elbow FE in °	0.072	**0.000**	0.0	**0.008**	**0.005**
	Shoulder FE in °	0.023	0.036	**0.008**	0.0	0.016
	Shoulder IER in °	0.134	0.020	0.003	0.008	0.0
	UE-FMA total	**0.002**	**0.008**	**0.001**	**0.001**	**0.000**
	UE-FMA subscore A	**0.004**	**0.005**	**0.000**	**0.000**	**0.000**
	UE-FMA subscore B	0.063	0.443	0.663	0.162	0.068
	UE-FMA subscore C	0.625	0.860	0.626	0.627	0.077
	UE-FMA subscore D	**0.007**	**0.006**	**0.001**	**0.008**	**0.001**

*Indicates significant correlations at the p < 0.01 level. Displayed is the cross-correlation of the UE-FMA score, the UE-FMA subscores A (upper extremity, max. 36 points), B (wrist function, max. 16 points), C (hand function, max. 14 points) and D (coordination/speed, max. 4 points). *FE* flexion/extension, *IER* internal/external rotation

A multiple regression analysis was performed to predict the UE-FMA score from grip pressure, wrist FE, elbow FE, shoulder FE, and shoulder IER. Linearity was assessed by partial regression plots and a plot of studentized residuals against the predicted values. Independence of residuals was assessed by a Durbin-Watson statistic of 1.97. Homoscedasticity was assessed by visual inspection of a plot of studentized residuals versus unstandardized predicted values. Since none of the tolerance values exceeded 0.1, there was no evidence of multicollinearity. No studentized deleted residuals were found to be greater than ± 3 standard deviations, no leverage values were greater than 0.2, and the values for Cook’s distance were above 1. As assessed by a Q-Q Plot, the assumption of normality was confirmed. A multiple regression model predicted the UE-FMA significantly (F (5, 13) = 12.22, p < 0.0005, adj. R² = 0.83). Shoulder IER and grip pressure, with standardized coefficients β of 0.55 and 0.38, respectively, both added significantly (p < 0.05) to the prediction (Table 3). In the post hoc power analysis [41], the predicted multiple regression model (p < 0.001, df = 5 and n = 19) had a statistical power between 0.83 and 0.89.

**Table 3:** Summary of multiple regression analysis

Variable	B	SEB	β
Intercept	4.341	1.670	
Grip pressure	0.253	0.90	0.380*
Wrist FE	− 0.009	0.056	− 0.027
Elbow FE	0.049	0.064	0.152
Shoulder FE	0.039	0.054	0.111
Shoulder IER	0.462	0.130	0.552*

B: unstandardized regression coefficient; SEB: standard error of the coefficient; β:  standardized coefficient; FE:  flexion/extension; IER: internal/external rotation. *p < 0.05

## Discussion

In this study, we used an exoskeleton-based assessment protocol to investigate the convergent validity of the acquired sensor-based data in comparison with the UE-FMA clinical outcome measure in severely impaired stroke patients. The UE-FMA was significantly predicted by the separately measured single-joint angles (with shoulder IER contributing most) and grip pressure.

This approach differed from most previous work in this field by applying a self-contained evaluation task that was separated from the rehabilitation exercises of the same device to avoid exercise-specific learning effects [34]. Thereby, the implemented evaluation task will provide a modular extension of the custom-made soft- and hardware applications and functionalities that have been already developed for the same exoskeleton framework: e.g., online feedback of extent of movement and quality for the assisted ADL-like exercises [9, 37], closed-loop task difficulty adaptation of these virtual reach-to-grasp tasks [8], and hybrid exoskeletons including adaptive neuromuscular stimulation [11], additional brain control [12] and robotic support with active actuation [20].

We assessed the single-joint angles in a standardized fashion that is often applied in the clinical context of neurological and orthopedic evaluations (i.e., beginning from the normal zero position with 90-degree distance between forearm and upper arm). This distinguishes our approach from previous methods for kinematic movement assessment that were acquired during specific exercises with more complex movements [42]. Therefore, the algorithm proposed in this study can be transferred to those measurement systems that allow the registration of different joints. This approach would, therefore, provide a standardized assessment that could be performed on different devices and enable better comparisons of the studies. However, it should be noted that different measurement systems (mechanical vs. optical, exoskeleton vs. endpoint -based) will differently influence, e.g., friction, inertia and arm weight support, and would need direct comparisons before further conclusions may be drawn.

The patients in this study were so severely impaired that the range of motion of the exoskeleton was not exceeded; therefore, no saturation occurred. Previous studies, where healthy subjects performed activities of daily living with this exoskeleton, also showed no limitation of the range of motion [8, 9]. However, when comparing the physiological grip strength [39] and active range of motion [40] of healthy subjects with the device maxima (all indicated in the caption of Fig. 1), elbow FE (146° vs. 90°) and shoulder FE (158° vs. 90°) may exceed the range of motion of the exoskeleton. This may become relevant when investigating less severely impaired patients with this approach. Furthermore, the glenohumeral joint of the shoulder has three degrees of freedom with largely coupled range of motions [43], while our assessment system could sufficiently assess only two of them.

To increase the assessment frequency in the clinical environment [6], the assessment time is critical. The positioning of the patient in the exoskeleton (~ 5 min) and the movement assessment (~ 6 min) were relevantly quicker than the clinical evaluation with the UE-FMA score (~ 30 min). Since this kinematic evaluation will be done in centers that use the exoskeleton for training purposes anyway, we would recommend doing the assessment either before or after the respective training sessions to track improvements in the course of a rehabilitation period; thereby, an addition positioning of the patient in the exoskeleton will be avoided and the time savings in comparison to the clinical evaluation even further improved. However, future studies need to investigate the inter- and intraoperator reliability of different therapists applying this protocol.

This has already been done for the clinical UE-FMA score [31], thereby, demonstrating excellent inter- and intrarater reliability and responsiveness to changes of motor impairment [44]. However, fine distal motor functions may be underrepresented in the UE-FMA scale, and a ceiling effect of the motor function has been reported [45]. In this context, the convergent validity with the UE-FMA suggests the use of exoskeleton-based measurements for a finer and more specific registration of movements [30].

Furthermore, the UE-FMA is relatively time-consuming and cannot be performed in the context of an exercise session, e.g., to track the improvement during a training period. With the assessment task presented here, detailed information on progress at the impairment level can be provided before and/or after each training session with minimal additional time. It is important that this information is not influenced by training exercise-specific effects of the actual therapy. The task should be self-contained and only aim at motor assessment [29, 34].

The implemented setup contained an integrated virtual reality module to provide immediate and continuous feedback of the movement extent [37, 46–48]. Such approaches are important to motor learning in rehabilitation [8, 9, 49] and have been expanded here to the area of instrumental assessment. However, the basic concept of exoskeleton-based single-joint assessment does not depend on this virtual reality feature or the complexity of the applied upper limb model. Future studies need to investigate the added benefit of these additional components.

Range of motion evaluations in comparison to clinical outcome measures after stroke have previously been performed. However, these studies were not conducted in severely impaired patients who were unable to perform these movements without gravity-balancing and/or did not apply self-contained single-joint assessments [15–17, 33, 50, 51]. In these previous studies, often optical motion tracking systems or inertial sensors were used during reach-to-grasp exercises (which the patients could perform without external support), and the angle data was extracted subsequently from the movement trajectories. The respective findings in these less impaired patients should, therefore, only cautiously be compared with the present work:

Cristea et al. [33] found the level of motor function to be significantly correlated with three kinematic measures: elbow extension (r = 0.81), shoulder flexion (r = 0.89) and trunk displacement (r = − 0.86). Michaelsen et al. [16] reported correlations with wrist extension (r = 0.46) and hand function (r = 0.54), but not with grip strength. Meulen et al. [15] identified correlations with the range of vertical hand elevation (r = 0.66), a movement parameter composed of elbow and shoulder flexion. Beebe et al. [17] consecutively evaluating the active range of motion of shoulder, elbow, forearm, wrist and fingers, and performed a comparison with different clinical tests that were integrated into one score for upper limb function using a principal component analysis. Thereby, the highest correlation was found for shoulder IER (r = 0.81). However, no UE-FMA score was used in this study.

In this context, the choice of task, measurement system and metrics in the present study addressed first and foremost the impairment level of severely affected patients who were unable to perform movements without gravity-balancing. We demonstrated that shoulder IER, a frequently neglected proximal component of kinematic assessments of the upper limb [30], was the measure with the most relevant contribution to the prediction of the UE-FMA scale. This may in part be related to the over-representation of the shoulder in the different UE-FMA evaluation tasks [45]. Notably, there was only a weak correlation of the measured wrist angle and grip pressure with the UE-FMA subscores for wrist and hand. The more complex functional tasks required to perform the UE-FMA subscores seem not to be adequately represented in the simpler instrumental measures used here. However, the grip force, which tends to be under-represented in the UE-FMA, predicted the clinical status better than the joint movements (apart from the shoulder IER). This finding may suggest that this fairly straightforward instrumental measure—which can be easily acquired even without an exoskeleton—may be best suited for a clinically relevant and practical quantification in a potentially wide variety of patients after stroke. Importantly, future work needs to study larger, more heterogenous and independent sample sizes to confirm the predictive properties of the investigated kinematic parameters.

## Conclusions

In conclusion, an exoskeleton-based assessment of single-joint angles facilitated the rapid evaluation of the upper limb range of motion in severely impaired stroke patients with high convergent validity. Shoulder internal/external rotation contributed most relevantly to the prediction of the clinical status.

## Data Availability

The datasets are available from the corresponding author on reasonable request.
